# ‘It is a bloody big and responsible job’: perspectives on climate change communication from Australia-focused practitioners

**DOI:** 10.1007/s44168-022-00021-6

**Published:** 2022-08-19

**Authors:** Nicholas Badullovich

**Affiliations:** 1 Crawford School of Public Policy, Australian National University, Canberra, ACT Australia; 2 Institute for Climate, Energy & Disaster Solutions, Australian National University, Canberra, ACT Australia

**Keywords:** Climate change communication, Communication practitioners, Thematic analysis, Qualitative interviews, Climate-change policy

## Abstract

Climate change is a complex social-political issue in Australia with a history of dysfunctional conflict over climate policy. This presents challenges for the practitioners communicating about climate change to different publics. To date, academic research has focused on exploring how effective communication can be achieved but rarely has this been understood from a practitioner’s perspective. I present the findings of nineteen (*n* = 19) semi-structured interviews with climate change communication (CCC) practitioners in Australia. The interviews explored occupational experiences as a means to understand the state of practice, challenges, and ways forward for CCC. I explore four key themes in this analysis: the practitioners, their goals, the barriers, and their perspectives on framing. Participants spoke of the complexity in doing CCC work, including many barriers, but their focus on effective communication and engagement remains strong in the face of challenging circumstances.

**Graphical abstract:**

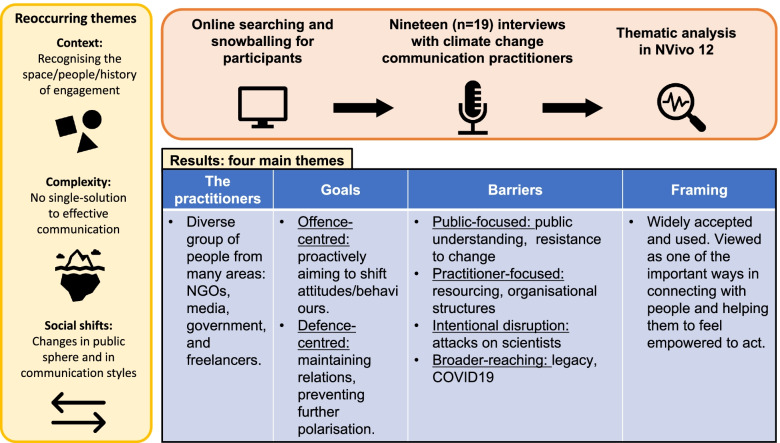

## Introduction

In Australia, the issue of climate change has been characterised by a history of social conflict and a legacy of complex and often failed policy responses at the national level (Christoff 2013; Crowley 2017, 2021). History has shown steps in the right and wrong directions as shown by Talberg et al. (2013) with Australia creating the first greenhouse gas reduction-focused government agency while also (around late 2013) repealing other emissions reduction legislation. A structural reliance on fossil fuels as well as successful lobbying have been identified as factors affecting the formation of climate policy in Australia (Crowley 2021). This has been the reality for federal policy despite public opinion which suggests a majority of Australians support climate policy with more than 80% of citizens wanting to see an orderly phase out of coal (Quicke 2021). Implications of this history can be seen in the public sphere, with social attitudes towards climate change in Australia becoming polarised along political lines, reflecting patterns observed in the USA, though evidently to a lesser magnitude (Hornsey et al. 2018). Recent research shows that while climate policy has social support in Australia, its importance to voters varies across political party preference (Colvin and Jotzo 2021) at least at the time of the 2019 Australian federal election. These social and political differences — often identity driven (Bliuc et al. 2015) — make climate change an extremely complex social-political issue within the context of Australia.

One area of focus in academic research and in practice that seeks to disentangle the social complexity of climate change has been on improving how climate change is communicated. The communication of climate change has been recognised as increasingly important for ensuring a broad social support base can be built, leading to the longevity of policy solutions (Corner and Clarke 2017; Romsdahl et al. 2018). Research broadly focusing on the communication of climate change involves a wide variety of fields and disciplines such as media studies, psychology, and both political and science communication (Fielding et al. 2014; Kahan 2015; Schäfer 2015). A climate change communication focus has become so prominent in academic research (Corner and Clarke 2017) that it is now arguably becoming a distinct subtopic of environmental communication scholarship (Comfort and Park 2018).

Academic research has continued to contribute to our understanding of how to communicate climate change, although there has been a lack of attention given to the people doing this communication work — climate change communication (CCC) practitioners. Previous studies have provided deep and rich insight into the experience of distinct groups of communicators covering climate change such as journalists (e.g. Gibson et al. 2016; Schäfer and Painter 2020). However, while journalists are important communicators (Eide and Kunelius 2016), it is also important to explore the practitioners as a whole, being inclusive of different working professions. There has been a general lack of research attention given to practitioners of climate change communication with a focus in academic literature being more centred around exploring the communication itself. This is potentially problematic as some scholars have noted a growing gap between CCC research and practice (Han and Stenhouse 2015).

This study presents an exploration of the people doing climate change communication work in Australia and their practices. Through qualitatively engaging with the experiences of CCC practitioners, this study aims to provide insight into their daily practices, thereby putting a much-needed focus on the people engaged in this work. This study presents findings from nineteen semi-structured interviews with CCC practitioners prominent within the Australian climate policy discussion and/or public sphere, analysed using a reflexive thematic analysis approach (Braun and Clarke 2022).

## Background

Climate change is not simply a scientific issue but one that has inherent links to society. It can be thought of as a cultural object which demands an understanding of the social world (Hulme 2008). This matters when considering the communication of climate change, especially if taking the perspective of communication being ritualistic (Carey 2009). In other words, a model of communication that is different to traditional transmission (see Irwin and Wynne 1996; Ockwell et al. 2009) which instead represents a richer social experience reconciling constructs like values and identities (Corner and Clarke 2017). Climate change communication itself is a rich area of research evidenced in part through the diversity in topics such as exploring effective communication frames (Rode et al. 2021; Stanley et al. 2021b), trends in global media (e.g. Hase et al. 2021; Vu et al. 2019), and visual communication (e.g. Altinay 2017; O’Neill and Smith 2014) to name a few.

A common focus for CCC research has centred around the role of communication itself and understanding factors that can improve or inhibit it. This has left a noticeable gap in our understanding of ‘the people’, contributing in part to a gap between scholarship and practice. The lack of formal recognition for academics engaging in practical work and the inaccessibility of research for practitioners are factors that could contribute to this gap, though systematic evidence of this phenomenon lacks (Moser 2016). Han and Stenhouse (2015) argue this research-practice gap already exists and identified the importance of addressing it through researcher-practitioner collaborations.

### People currently communicating climate change

Climate change communication practitioners are not clearly defined in the academic literature. An audit survey of UK-based CCC practitioners by Mcloughlin et al. (2018) found a mix of people from research organisations, nongovernment organisations (NGOs), government, and media among others. Similar findings were identified by Rohling et al. (2016), although in a US context and their participants (climate change communicators) represented areas such as universities, government agencies, and NGOs. Körfgen et al. (2019) aimed to understand the status quo of CCC in Austria, and participants in their study represented stakeholders from civil society, churches, and the media (among others). Considering the barriers to practice, UK-based climate change communicators face challenges such as communication events/activities not meeting audience needs and a lack of post-activity evaluation (McLoughlin et al. 2018). Similarly, Körfgen et al. (2019) identified challenges like neglected audiences/topics, challenges in messaging, and difficulties dealing with uncertainty.

Experiences can vary for different sub-groups of communication practitioners. For example, studies exist which look at the CCC role of the following: journalists (e.g. Gibson et al. 2016), NGOs (e.g. Lück et al. 2016), celebrities (e.g. Doyle et al. 2017), and meteorologists (e.g. McIlroy-Young and Thistlethwaite 2019). It would not be possible to explore all the above occupations in this paper, although looking at the experiences of journalists is a useful group for gaining additional background. Furthermore, there exist numerous studies with journalists covering climate change as the focus.

Journalists play an important role in the communication of climate change (Hackett et al. 2017) as they can influence perceptions of scientific uncertainty (Dunwoody 1999), and the media is one of the dominant sources of climate information for people globally (Schäfer and Painter 2020). In terms of covering climate change, there is no defined ‘beat’ where climate change fits; it crosscuts many journalistic areas which was identified as a challenge for some journalists covering climate change (Brüggemann 2017). Furthermore, there have been notable role shifts over time in practice such as a change from adhering to the journalistic norm of balanced reporting to a more ‘weight of evidence’ approach (M. T. Boykoff and Boykoff 2007; Brüggemann and Engesser 2017; Hiles and Hinnant 2014; Kohl et al. 2016) and changes in coverage from episodic to routine. Gibson et al. (2016, p. 428) highlighted through interviews with environmental journalists some challenges of covering climate change such as its complex and global nature, thereby making it harder to engage people locally, and the fact it lacks a distinct ‘human face’.

### Framing in communication

The creation of communication can involve many considerations, one of which is how that communication is framed. Framing is a way of selecting certain elements of an issue and emphasising those over others in order to create tailored understandings (Corner and Clarke 2017; Entman 1993; Nisbet 2009). Many disciplines have contributed to framing which has both advanced conceptual richness but also vagueness as argued by some (Entman 1993; Scheufele and Iyengar 2014). Given the often-contested perspectives on framing in the academic literature, this analysis was guided in part by framing theory to help enrich the discussion from a practitioner perspective.

Climate change framing research comprises of two general foci: the first being understanding how communication is framed and the other being the effects of those frames on people and their understandings. However, one notable critique of the framing literature is its positioning within controlled laboratory environments, thereby leading to lack of confidence in framing effects outside of these settings (Druckman and Nelson 2003; Goldberg et al. 2020). Furthermore, people are bombarded with many frames everyday which can compete for attention known as frame competition (Amsalem and Zoizner 2020). Hence, it could be argued that there exists a tension between framing research and how that it is applied in practice. Therefore, gaining practitioner perspectives on framing may help highlight its function in practical communication on climate change.

## Materials and methods

Using semi-structured interviews, I engaged with Australia-focused CCC practitioners to learn about their experiences, and this was guided by some key research objectives (RO):

- Who is currently engaging in CCC work? (RO1)
- Are there any barriers faced by practitioners, and, if so, what are they? (RO2)
- What is the role of framing in CCC? (RO3)

The interview guide was developed to include a wide variety of questions and trialled informally for refinement. Questions were adjusted until the final guide was created. The project was granted ethical clearance by the Australian National University’s Human Research Ethics Committee (protocol number: 2020/697). Participant recruitment began with organisation searching online and then proceeded with snowballing.

In total, nineteen (*n* = 19) semi-structured interviews were conducted (mostly online), and they typically lasted between 25 and 75 minutes with average time being around 50 minutes. Given many people could realistically participate in CCC, boundaries needed to be drawn around participant eligibility to limit the scope. For this reason, it was decided that a participant needed to be conducting CCC as a core part of their occupation. In other words, people engaged in communication in their personal time were excluded, as well as academics doing communication research (this enabled the focus to be predominately in the practical and not academic sphere). Participants (see Table 1) varied in their working experience and qualifications with some having decades of experiences and others being earlier in their careers. Backgrounds varied for participants as some came from previous scientific careers, while others had more communication-focused backgrounds and training.

**Table 1:** General information about the participants across different attributes

Attribute	No. of participants
Working area (*n* = 19)	
Government	6
Independent	3
NGOs and advocacy	8
University	2
Gender^a^ (*n* = 19)	
Female	13
Male	6
Age^a^ (*n* = 19)	
Under 50	15
Over 50	4

^a^These attributes were not directly collected from the participants but instead inferred by the researcher

Interviews were transcribed and then analysed in NVivo 12 guided by a reflexive thematic analysis (Braun and Clarke 2006, 2022). Thematic analysis was suited as it allows for an inductive analysis approach while being flexible enough to incorporate various guiding frameworks like framing theory. Both semantic (inferred explicitly from the transcripts) and latent (interpreted from the transcripts) interpretation (Braun and Clarke 2022) was used, providing a grounding in the dataset while also putting these data into conceptual typologies. A social constructivist perspective guided this research and analysis which acknowledges that individual and subjective factors shape reality and how it is understood (Moon and Blackman 2014). In this case, the values, experiences, knowledges, and perspectives of the participants would all factor into their responses, as well as the positionality of the researcher. This positionality was appropriate as the diversity in participant occupation makes it difficult and arguably meaningless to justify a single shared reality or ontology. Multiple passes of the dataset were conducted during the coding phase in order to develop and refine the themes. Quotes from participants are provided in the analysis section, and the use of “[…]” indicates editing of a quote for the purposes of parsimony while highlighting the main message.

## Analysis and discussion

The following section will present an integration of results and discussion following the thematic analysis. It would not be possible to explore all themes present in this analysis; hence, four key themes will be discussed: *the practitioners*, *goals*, *present barriers*, and *the use of framing*. These themes were identified as being important in both addressing the research objectives and painting a rich picture of the state of CCC practice in Australia.

Before exploring each of the four themes, three crosscutting concepts were identified. These three crosscutting concepts are relevant to all themes, and it is therefore necessary to briefly describe them before presenting the rest of the analysis.

1. *Context*: Context arose as one of the most prominent and important concepts from the data. In this piece, context describes the idea that effective CCC work cannot be conceptualised, carried out, or understood without an understanding of contextual factors. When trying to understand context, one may consider the specific audiences or publics and their sets of values or beliefs. Other considerations may be specific geographical regions or even recognition of the history that an area has with respect to previous climate change engagement. Context is both an overarching concept and a recommendation, meaning that context should be an important consideration when carrying out climate change communication practice, as well as when conducting research.

That’s another thing about climate change communication, there’s no one-size fits all, it’s all very context dependent: who are you trying to communicate to and why are you trying to communicate that? That really changes how you communicate it (IWC11)

(2) *Complexity*: The complexity of climate change arose as one of the specific barriers to communication practice (described below); however, the idea can be broadened to recognise the fact that climate change complexity is not inherently negative. It then follows that communication work on a complex topic may also be a complex practice (Corner and Clarke 2017), involving many variables such as consideration of audience, balancing different goals, and deciding what ‘should’ be communicated (i.e. being responsive to context). Complexity is the recognition that CCC, much like climate change itself, is multi-faceted and is not a single-solution problem. Furthermore, effective CCC is not a case of finding a silver bullet (Moser 2016), nor was that a belief shared by the participants from this study. This further highlights the ability of CCC practitioners to work within a complex space and navigate various issues to achieve their goals.

It’s in this weird space of the facts are clear but you can’t communicate them as if that’s always the case, you have to consider people’s different political views, personal views and account for those… (IWC11)

The politics around climate are so vexed and so many seemingly logical and rational and strategic communications and campaigns have failed in this space which just adds further complexity to figuring out how to communicate and how to influence change. (IWC4)

(3) *Social shifts*: Attitudes and behaviours change over time, as do the social and political structures that exist around climate change. Recent work has demonstrated this (Quicke 2021), and the future of climate change attitudes is also set to change (Colvin and Jotzo 2021). Similar trends have been observed elsewhere like in the USA despite the fact it is one of the most politically polarised countries on the issue of climate change (Hornsey et al. 2018; Leiserowitz et al. 2021)⁠. Hence, social shifts do occur, and flow-on effects exist for how CCC practitioners then conduct their work. For example, they cannot continue using outdated media (e.g. printed news) if their audiences are accessing their information through new media (e.g. Twitter, TikTok, and Snapchat). This overarching concept is comprised of two primary components where one is about the social/attitudinal/behavioural shifts that can occur for people. The second represents the shifts in attitudes and perceptions of the practitioners themselves. In some ways, this represents a practitioner reflexivity, a constant process of adjustment and refinement that practitioners engage in to ensure their work is relevant and impactful.

Climate change has moved out of the realm of just being for climate scientists… into the realm of really broad sub-section of society talking about it… we are past the tipping point of cultural change when you see things popping up in all of the forums (IWC14)

### The practitioners

I engage with climate change in a personal capacity as well as a professional capacity […] and I think there is a role for that because I think sometimes having personal opinions and personal stories on climate change can be more convincing than just data (IWC11)

A logical starting point in understanding the experiences of CCC practitioners in Australia is by understanding the people themselves. Participants worked in areas such as government, NGOs/advocacy organisations, in the university sector (though not as academics), the media, and some being independents/consultants. This provides insight into RO1 and confirms previous work that CCC practitioners are a nonhomogeneous group of people coming from a variety of occupations (Körfgen et al. 2019; Mcloughlin et al. 2018; Rohling et al. 2016). Results from this research suggest climate communication work in Australia is better defined as a practice (as opposed to a discrete occupation) which involves people from diverse occupations.

Science communicators are not just a profession. There are thousands of different people involved in science communication. Scientists, teachers, journalists, bankers. Science communicators exist in every profession (IWC6)

Qualities such as authenticity and credibility were discussed as being important factors that a communicator must embody. To be authentic in communication was depicted as a communicator being ‘real’. This meant the perception of whether a communicator came across as ‘real’ depended on the specific public being engaged. Furthermore, as the quote at the beginning suggests, qualities help to highlight the human angle and the personal (as well as professional) motivations the practitioners had with their work.

Messengers who genuinely embody those core attributes we talked about before of authenticity, credibility and trustworthiness, then they can come from all walks of life and still be incredibly effective (IWC18)

As mentioned above, the perception of authenticity depends in part on the public being engaged. One communicator may not carry the same level of perceived authenticity or credibility if a public sees them to be different from themselves (Sparkman and Attari 2020). This can be linked with the idea of an ingroup messenger as described in the social identity approach (Fielding et al. 2020; Haslam 2004). Ingroup messengers can be influential in their communication due to the levels of trust and credibility they can establish with their publics, provided those publics perceive the messenger as part of the ‘ingroup’ (Hornsey et al. 2002). Fielding et al. (2020) demonstrated this by showing ingroup messengers carried greater persuasive effect when communicating about climate policy to their specific publics, in this case US political parties (see also Goldberg et al. 2021).

Advocacy or ‘being an advocate’ was a discussion that revealed a tension in the dataset. Discussions around the relationship between ‘being a scientist’ and ‘being an advocate’ are present in the literature (Boykoff and Oonk 2020; Nelson and Vucetich 2009). However, in the context of this study, discussions were centred around whether CCC practitioners are advocates for climate change action (e.g. encouraging behavioural change or policy support) or not. Some participants were embedded in advocacy-focused organisations, while others held non-advocacy-focused roles. One participant from an advocacy-focused organisation made clear the role that advocacy played in their work.

At least for the past kind of 10 years, we have been very advocacy focused (IWC17)

In contrast, a contrary position was also highlighted by a participant working within the government, and that advocating was not part of what they do in their occupational work. In this case, context (particularly that in government) was drawn upon to help and establish this position as being an impartial informer.

And really, when we’re communicating, it’s really making sure it’s giving that sense that we’re not lobbyists; we’re not advocates for a particular cause; we are doing broad stakeholder engagement, looking at the data and coming up with recommendations (IWC3)

Interestingly, a position in between these two was raised by another participant also embedded in a government context. Interpretation of latent themes suggests some amount of reflexive thinking going on and may represent an occurring social shift.

I think that we [CCC practitioners] are perceived a bit as issue advocates rather than analysts or people providing […] sort of disinterested analysis. Which is unfortunate, but I […] frankly think it’s kind of true (IWC19)

What is made clear is that the position of being an advocate is not only unclear with respect to scientists but also CCC practitioners. Being an advocate was a label that some, but not others, were comfortable using. While advocacy may seem an inherent part of communicating about climate change, it is clear a tension exists around the conceptualisation of advocacy and an awareness of the connotations if deciding to use the label of ‘advocate’ (Boykoff and Oonk 2020).

Participants in this study were diverse CCC practitioners, though they provided strikingly similar answers to the ‘responsibility’ question: ‘who (in addition to themselves) *should* be communicating climate change?’ Reponses to this question tended to fall into one of two groups: (1) participants would list many different people and occupations to emphasise the importance of diverse communicators.

Parents need to be communicating about climate change. […] parents and teachers, and the government, I guess, needs to be communicating climate change […] politicians as well should be communicating about climate change … and other influencers as well, I think. Sports people (IWC1)

The second (2) kind of response from participations to this question was ‘everybody’, without differentiating by social role or occupation.

Everyone. Everyone should be communicating. […] It doesn’t really matter how you do it but everyone should be doing it. (IWC15)

Everyone should be a climate action communicator, I think that's really important. (IWC5)

Everybody is a science communicator, potentially. (IWC6)

The identification from practitioners that CCC is something everyone can and should do sheds some light onto the evolving nature of climate change. Early days of CCC which used mainly deficit style communication had a focus on scientists providing credible information (Moser 2010; Nerlich et al. 2010). Nowadays, like science communication, CCC is no longer only rooted in deficit-style thinking and involves understanding the communication as well as the people and how climate change interacts with society (Ballantyne 2016; Trench and Bucchi 2010). What the response above suggest is that there may be a shifting role of the CCC practitioner. Originally being the ‘source’ of communication — as Nerlich et al. (2010) argued, it was in the beginning under the ‘public understanding of science’ model — to empowering people with the tools to facilitate their own communication in interpersonal settings. This is consistent with contemporary conceptualisations of climate change engagement (see Corner and Clarke 2017).

At the end of the day I think my goal is to give the power back as much as possible, and that means giving people space to have a voice heard, but it also means giving people faith in their own power (IWC18)

### Goals

I just think we need to mainstream discussion of climate change. […] and to do that, we've got to empower all these people and kind of bring people and move people along that kind of spectrum (IWC2)

Goals are an important part of communication, particularly in the context of overarching strategies. Goals ranged from being very precise (e.g. reaching a specific audience group) to being broader in scope (e.g. normalising conversation) which typically take a longer time with sustained efforts (Badullovich 2022; McAlevey 2016). Overall, three different types of goals emerged from this analysis, and they map to both strategy and tactics. Conceptually, strategy tends to act as a guiding framework towards a single or set of objectives with tactics being specific activities implemented ‘on the ground’ to achieve that (Han and Stenhouse 2015; Wilcox et al. 2017). The CCC practitioners spoke of both strategy and tactics-related goals which have been summarised in Table 2. Fewer participant quotes are drawn upon in this section as this part of the analysis involved more grouping of ideas to come up with a latent description of the different kinds of goals present in CCC practice. The analysis in this section is the result of integration across many different sub-nodes, and semantic goals mentioned the interviews.

**Table 2:** Strategy and tactics-related goals of CCC practice

Goal type	Description	Example
Strategy: broader social change	Focused on the broader and less-tangible outcomes of public engagement with climate change. They are typically broader in scope and require sustained efforts in achieving. Additionally, the direct outcomes of these goals may not be immediately recognisable, but they are important in overall strategy	‘Yeah, well I guess for me the broad goals are really to actually just get people to take action that contributes to the broader whole because it’s about individually what we can do to collectively achieve those actions’ (IWC13)
Tactics: offence centred	Goals are designed along with communication interventions to maximise persuasive effects and typically focus around bringing about desirable shifts in attitudes and behaviours	‘It’s more about empowering our supporters to take action rather than actually lobbying someone over Twitter because that would never work’ (IWC14)
Tactics: defence centred	Communications are designed to prevent further dysfunction or polarisation. The intended outcome is a repair of the social relationships or at least prevent a further polarisation of attitudes	‘We don’t want to unnecessarily ostracise people from the climate change […] field, we want to include as many people as possible’ (IWC11)

‘Strategy related’ pertains to broader social changes that are looking to be achieved. These strategy-related goals are more general and help to shape the nature of tactics. ‘Tactics-related’ goals are termed as such because they sit in more ‘on the ground’ contexts. Two main tactics-related goals were identified, and these were offence centred and defence centred. Offence-centred goals are conceptualised as communication that is designed to help shift attitudes or encourage specific behaviours through creating awareness and empowerment. The defence-centred goals have a different purpose where the main motivation is to prevent a deepening of social division. Put another way, defence-centred goals are about preventing negative outcomes and maintaining or repairing social relationships. Specific examples of tactics-related goals (that were coded as nodes) were as follows: *raising/creating climate change awareness*, *encouraging climate action*, and *influencing elites*.

### The barriers

We are here to change how humans think about now and the future, the present and the future. So it’s a bloody big and responsible job (IWC6)

A critical element in understanding the state of practice of CCC in Australia requires exploration of the strategies as well as the difficulties. Participants were asked if any barriers exist in their practice, and the insight helped to address RO2. The participants spoke at length and in detail about the kinds of barriers they face in their work. ‘Barriers’ were the most highly referenced code with over 300 coded passages and more than 60 different barriers or challenges mentioned. However, it is worth noting the goal here is not to quantify the number of barriers; instead, giving a numerical indication here is to demonstrate the vastness of the discussions around barriers in CCC work. This section will present a collation of these barriers into five key subthemes under the general barriers theme.

When asked if barriers existed in their practice, participants responded overwhelmingly with numerous barriers and difficulties. A barrier, in this study, was defined as a situation, object, or factor which can present challenges to carrying out CCC work in the intended way, although not necessarily negative. Barriers are an encompassing term and can range from being very minor and specific to one context, to being very broad and pervasive. Both semantic and latent barriers were present (Braun and Clarke 2022). Table 3 presents a collation of the barriers into key subthemes. In the case of ‘context specific’, this is a manifestation of the pervasive cross-cutting *context* concept. Some barriers are only experienced in certain occupations and not others which suggests barriers should be understood for each occupation and not generally across different CCC settings.

**Table 3:** Collation of the barriers to CCC work discussed by practitioners

Barrier subtheme	Description	Examples
Context specific: this is a condition that broadly represents the idea that some occupations or communication environments will have specific barriers because of their organisational structures or features of the type of communication work that is undertaken. Examples from the media profession are as follows: *changing media landscape*, *pressure on journalists*, and *constraints on writing*		
Public focused	The public-focused barriers are those that are typically conceptualised in the context of CCC work. They are barriers that focus on the challenges working with specific audiences or publics and could be considered the most ‘visible’ barriers	*People being resistant to change*, *being time poor*, and *public understanding of climate change*
Practitioner focused	Practitioner-focused barriers represent challenges which are not necessarily the result of publics or audiences. These barriers represent the personal and occupational challenges that can arise during CCC work. They are less known in the sense that they might not be considered a ‘traditional’ barrier because they are not audience focused	*Resources (funding)*, *juggling many responsibilities*, and *measuring impact*
Intentional disruption	Situated underneath public-focused barriers, the intentional disruption sub-theme captures the fact that some people/groups purposely try to disrupt climate communication or action	*Attacks on scientists*, *delaying tactics*, *vested interests*
Broader reaching	Broader reaching barriers are those that tend to be more pervasive in nature. They do not necessarily crosscut all other subthemes; however, their general nature means that they can be present in many practical contexts	*Legacy*, *facts and values*, *COVID-19*

Barriers to conducting climate change communication can come in many forms and are arguably the result of the inherent complexity of the issue. The barriers identified in previous studies tend to capture those relevant to the public- and practitioner-focused sub-groups (Körfgen et al. 2019; Mcloughlin et al. 2018). Specific barriers pertaining to certain occupational environments have also been documented by McIlroy-Young and Thistlethwaite (2019) who find that Canadian meteorologists in the government context are more unwilling to make connections between local weather and climate change due to potential political ramifications, compared with other private or public broadcasters. This demonstrates the idea that challenges to CCC practice are not ubiquitous across all communication environments, and hence, the *context-specific* contextual factor is a condition that sits around the suite of barriers identified in this research. Some practitioner-focused barriers reflect personal shortcomings; however, many of these barriers appear to be the result of complexity with climate change being a global issue involving varying values and identities. A final noteworthy point is that *intentional disruption* barriers were not frequently spoken about, suggesting CCC practitioners are perhaps not as readily engaging with people sceptical/denialistic about climate change. However, the drivers behind this were not discussed, although one potential explanation is that the portion of people denying the existence of climate change is relatively small (compared to those accepting the scientific evidence) in Australia (Quicke 2021).

### The use of framing

Any kind of engagement with the community, anything where we’re trying to change anyone’s behaviour, we should be thinking about framing and we should be thinking about what’s important for them. (IWC13)

Part of the interviews involved discussions around the concept of framing and how it applies in CCC practice. As mentioned in the background, framing is a common way of tailoring communication to specific groups, however is generally contested and critiqued as an approach for effective communication in academic literature (Chong and Druckman 2007; Rode et al. 2021). Gaining the framing perspectives of practitioners (addressing RO3) could help in ensuring future framing work is best oriented to support communication practitioners.

All participants generally regarded framing as an important component during communication, and scholars have suggested communication itself is never without a frame (Nisbet 2009). Entman (1993) argued that frames in communication can reside in at least four places: in the text, with the communicator, with the receiver, and in culture. In many cases, frames were tied to the practitioners and their actions, although the use of frames tended to depend on the audience/public’s needs.

Framing is pretty much everything; it’s very important. Yes, I do use it; […] for example, how we get the key message across […] it all just comes down to framing; setting the context (IWC1)

Yeah, really strongly, we definitely use it in our work […] any kind of engagement with the community, anything where we’re trying to change anyone’s behaviour, we should be thinking about framing and we should be thinking about what’s important for them (IWC13)

The emphasis on the publics being engaged was consistent across discussions with the CCC practitioners. Frames were not spoken about as being objective devices that are the result of scientific truth. Put another way, a positivist view of framing (where the issue/evidence should dictate the frame) was not generally evident in the interviews; frames were always discussed as being dependant on the public being engaged. This extends on Schäfer and O’Neill (2017) who argue that framing is a constructivist concept.

So, I think framing is about thinking about the audience and how the audience receives messages, and planning of communication and the content of that communication with the audience in mind rather than what you want to say, I think that’s really what the framing is about (IWC16)

When discussing effective frames during communication, participants identified that including humans in the frame was important. This was situated within a broader discussion about the future of climate change communication and having ‘impacts on humans’ at the centre, especially when talking about related issues like energy transitions.

But in the past, when it came to the climate conversation, I think what you’d hear, is like, “we need to hear from people who suffer”. […] But the thing that I’ve been thinking about the most recently is actually hearing from the people who will face the changes of the energy transition directly. […] we need to talk about the people who are at the forefront of the energy transition, not just the impacts of climate change (IWC9)

The effectiveness of human-centred communication has been studied in academic research. One relevant dimension is the impact of climate change on public health which is intrinsically connected to people. Myers et al. (2012) found that a public health frame can reduce anger in ‘dismissive communities’ (see Leiserowitz et al. 2009) and lead to higher levels of hope. Additionally, emerging literature around ecological grief (see review from Ojala et al. 2021) has further highlighted the important role human emotion plays in communication (Stanley et al. 2021a, 2021b; Wang et al. 2018) and how some practitioners use this angle as their focus.

The discussion around framing was rich as it tapped into the motivations behind why CCC practitioners use frames. In some cases, framing was highlighted as just one potential tool or consideration, among others. For example, context was a critical factor in understanding how the practitioners conceptualised and used framing. In some circumstances, the participants spoke of how they do and do not use certain frames. In other words, there was time and consideration put in to determining which frames to use and which frames not to use following defence-centred goals in some cases.

Also avoiding […] terms like ‘climate change debate’, never framing it as something that’s still inconclusive or up for conversation. Saying climate change is a fact, never explicitly saying that, but communicating as if it is (IWC11)

Framing is typically conceptualised in academic literature as being a useful, albeit contested and complex communication tool for shifting attitudes on climate change (Chong and Druckman 2013; Druckman 2001). However, the participants in this study spoke of using framing not just for attitudinal shifts but also for facilitating productive discussion. This highlights the broader role framing appears to play in the work of CCC practitioners.

It can lead to other conversations with other people and discussions about different aspects of climate change. […] I think the way in which different dimensions of climate change are characterised can really change how receptive people are to engage in and talking about it. […] I think that communications and some of our events can actually cause people to appreciate and understand different aspects of the opportunities associated with climate change. And that might then spur them to think about discussing that with colleagues or with friends and family (IWC2)

### Limitations

Nineteen CCC practitioners were interviewed for this study making it difficult to generalise these findings to other CCC practitioners. However, it is worth mentioning that generalisation is not necessarily an appropriate concept when conducting qualitative research and thematic analysis (Braun and Clarke 2022; Kaya 2013) and was not a goal for this research. Quality is a more appropriate concept to judge a thematic analysis, and Braun and Clarke (2021) present tools for assessing this. This research was conducted with an awareness of how social and political factors will affect the experiences of practitioners and nature of the data. Therefore, it is more important to take these findings and view them within the relevant social-political context. Future research could conduct a similar exploration though in a different country or region and incorporate elements of comparison to determine the varying effects context could have on CCC practice.

Thinking about which groups to focus on for future research will be important. To have a sample of participants, there needed to be somewhat arbitrary boundaries drawn to act as inclusion criteria. Future research could go two ways from here: (1) step back and generate a detailed typology of different people communicating climate change (although as results in this study suggest that could simply be everyone) or (2) focus in on further exploration of specific sub-groups (e.g. CCC practitioners in government, media, or freelancers).

## Conclusions

This study has put a focus on climate change communication practitioners in Australia, with a goal of exploring their experiences in working with a highly complex social-political issue. While analysis of these interviews has uncovered a rich repository of CCC discussion, this work provides three main contributions:

1. Climate change communication is not a discrete occupation in Australia. Practitioners with varying backgrounds engage in this work in different occupational settings, with different goals, and face context-relevant barriers. This means the term ‘climate change communicator’ is best conceptualised as encompassing a wide cross-section of occupations in political, private, and public spheres. Hence, future research involving CCC practitioners should consider these diverse groups of occupations.
2. Climate change communication comes with a long list of barriers and difficulties. Public- and practitioner-focused barriers are common sub-groups, occupational context (in some ways) defining the relevant barriers, which means not every communication context will face the same difficulties. In the context of practice, this could mean relevant barriers should be identified so that communication can be best designed to overcome or work within them and be most effective.
3. The responsibility of communicating about climate change is not just on the practitioners but involves everyone. The participants spoke of the importance of everyone engaging in climate change conversations using public-relevant frames to ensure certain groups are not left behind. This speaks to importance of CCC practitioners not just communicating but helping people feel empowered to talk and act. For researchers, this provides a strong reason for investigating the role of conversation and discussion in communication settings.

While academic research has contributed extensively to our understanding of what does and does not work in CCC communication, insight has rarely come from a practitioner’s perspective. This paper helps in addressing the CCC research-practice gap by putting the focus on CCC practitioners and their experiences. It highlights the complex nature of communicating climate change and establishes some potential future pathways for exploring sub-groups of practitioners or specific goals or barriers. Climate change communication scholarship and practice should go hand in hand. Relationships and collaborations between researchers and practitioners will be critical for future efforts if we are to keep up with the ever-changing social context and ensure needs are being met on both sides.

## Data Availability

Data is not available publicly due to ethical considerations
